# 

**Published:** 1832-01

**Authors:** 


### LONDON MEDICAL AND PHYSICAL JOURNAL.

EDITED BY

**JOHN NORTH, Esq. F.L.S.**

MEMBER OF THE ROYAL COLLEGE OF SURGEONS, AND OF THE MEDICAL AND CHIRURGICAL SOCIETY OF LONDON;

AND

**GILBERT T. BURNETT, Esq.**

F.L.S. M.B. R.I. R.C.S. &c.

ROFESSOR OF BOTANY IN KING'S COLLEGE, LONDON, AND TO THE MEDICO-BOTANICAL SOCIETY.

(VOL. LXVII.)

NEW SERIES, VOL. XII.

*Et quoniam variant morbi, variabimus artes;*

*Mille mali species, mille salutis erunt.*

Surgeon Gen'l Office

45600

LONDON:

JOHN SOUTER, 73, ST. PAUL'S CHURCH-YARD;

AND TO BE HAD OF ALL MEDICAL BOOKSELLERS.

1832.

W1
LO 31

C. ADLARD, PRINTER, BARTHOLOMEW CLOSE.

### MONTHLY LIST OF MEDICAL BOOKS.

*[Medical Works cannot be entered on this List unless a copy be sent for the purpose, the titles of Books having frequently been sent to us as published which have not been published for weeks, or even months, after.]*

Cholera; its Nature, Cause, Treatment, and Prevention, clearly and concisely explained: with an Appendix, containing Practical Remarks on Fever and Dysentery, with which Cholera is intimately connected, and frequently combined; being the Substance of Reports made to the late Government of Poland. By Charles Searle, Esq., of the Hon. East India Company's Madras Establishment, and lately in charge of the principal Cholera Hospital at Warsaw. Second Edition, revised and enlarged.—8vo. pp. 51. Highley, London.

Memoir on Colouring Matter; to serve as an Example of the Manner in which Chemistry may be applied to the Arts, and to Investigations into the Nature and Properties of Organic and other aggregate Compounds: also an Essay on Light and Colour. By Edward Muston, Surgeon in the Service of the Honourable the East India Company.—8vo. pp. 119. Burgess and Hill, London.

Essays on the Effects of Iodine in Scrofulous Diseases; including an Inquiry into the Mode of Preparing Ioduretted Baths. Translated from the French of M. Lugol, Physician to the Hôpital St. Louis. By W. B. O'Shaughnessy, m.d. With an Appendix by the Translator, containing a Summary of Cases treated with Iodine, either Simple or combined with Opium, Mercury, or Lead; and Directions for Preparing the Iodurets of these Metals, and for detecting the Adulterations of Iodine and the Hydriodate of Potash.—8vo. pp. 218. Highley, London.

Part II. The Principles and Practice of Obstetric Medicine, in a Series of Systematic Dissertations on Midwifery and on the Diseases of Women and Children. Illustrated by numerous Plates. By David D. Davis, m.d. m.r.s.l., Professor of Midwifery in the University of London, &c.—4to. pp. 16. John Taylor, London.

On Indigestion and Costiveness; with Hints to both Sexes on the important, safe, and efficacious Means of relieving Diseases of the Digestive Organs by Lavements: including Directions for the Selection and Use of Apparatuses for their Administration, and the best Medicinal Preparations for Intestinal and other Injections. To which is added, Observations on the Mode of Preserving Health and Prolonging Life, by Air, Exercise, Sleep, Clothing, &c.: including many useful Family Prescriptions. The whole illustrated by coloured Engravings. By Edward Jukes, Surgeon, Inventor of the Stomach Pump.—8vo. pp. 196. Wilson, Royal Exchange.

How is the Cholera propagated? The Question considered, and some Facts stated. By an American Physician.—Pp. 22. John Miller, London.

Cholera; its Non-contagious Nature, and the best Means of arresting its Progress, shortly examined, in a Letter addressed to the Right Worshipful the Mayor of Newcastle. By T. M. Greenhow.—Newcastle.

Medical Case-book of Record, for Students and General Practitioners; with a Ledger for Use in Private Practice. By W. R. R. Wilton, Surgeon, &c.—Longman, London.

☞ A very convenient and well-arranged book for the purposes implied in the title.

An Address to the British Public on the late horrible System of Burking: containing an Account of the Methods hitherto adopted for supplying the Anatomical Schools with Subjects, and Suggestions for remedying the Evil, &c. By a Practical Anatomist.—Pp. 23. Renshaw and Rush, London.

☞ This is a very sensible letter, and we think that, if the suggestions thrown out by the writer were adopted, a complete stop would be put to the recent most horrible crimes, while, at the same time, anatomical schools would be sufficiently supplied with subjects, without wounding either the feelings of the public or of individuals.

An Essay on the Nature and Treatment of the Indian Pestilence, commonly called Cholera. By HENRY PENNECK, M.D.—Pp. 40. Highley, London.

Of Pestilential Cholera; its Nature, Prevention, and Curative Treatment. By JAMES COPLAND, M.D., Consulting Physician to Queen Charlotte's Lying-in Hospital, &c.— 8vo. pp. 174. Longman, London.

### NOTICES.

*We did not receive the account of the Lusus Naturæ from* Mr. OSWALD: *we shall be happy to insert it, if* Mr. O. *will favor us with it.*

